# Grape seed proanthocyanidin extract protects lymphocytes against histone-induced apoptosis

**DOI:** 10.7717/peerj.3108

**Published:** 2017-03-21

**Authors:** Ping Chang, Bing Mo, David M. Cauvi, Ying Yu, Zhenhui Guo, Jian Zhou, Qiong Huang, Qitao Yan, Guiming Chen, Zhanguo Liu

**Affiliations:** 1 Department of ICU, Zhujiang Hospital of Southern Medical University, Guangzhou, Guangdong, China; 2 Department of Surgery, University of California San Diego, La Jolla, CA, USA; 3 Department of MICU, General Hospital of Guangzhou Military Command, Guangzhou, Guangdong, China

**Keywords:** Grape seed proanthocyanidin extract, Lymphocyte apoptosis, Mitochondrial injury, Histones, Reactive oxygen species

## Abstract

Apoptosis of lymphocytes is associated with immunosuppression and poor prognosis in sepsis. Our previous report showed that histones, nuclear proteins released from damaged or dying cells in sepsis, can mediate lymphocyte apoptosis via mitochondria damage. Grape seed proanthocyanidin extract (GSPE), a natural substance with protective properties against oxidative stress, plays a vital role in cell and mitochondria protection. We thus hypothesized that GSPE may play a protective role in histone-induced lymphocyte apoptosis through its anti-oxidative properties. In this study, we investigated the protective efficacy of GSPE on lymphocyte apoptosis induced by extracellular histones, a main contributor of death in sepsis. Human blood lymphocytes were treated with 50 μg/ml histones, 2 μg/ml GSPE, or a combination of both. A total of 100 μM *N*-acetylcysteine (NAC), a reactive oxygen species (ROS) inhibitor, was used as a positive control for GSPE. Apoptosis, intracellular ROS levels, mitochondrial membrane potential, Bcl-2 expression, and caspase-3 cleavage were measured. Our data clearly indicate that GSPE significantly inhibited lymphocyte apoptosis, generation of ROS, the loss of mitochondrial membrane potential, the decrease in Bcl-2 expression, and caspase-3 activation induced by extracellular histones. In conclusion, we show that GSPE has a protective effect on lymphocyte apoptosis induced by extracellular histones. This study suggests GSPE as a potential therapeutic agent that could help reduce lymphocyte apoptosis, and thus the state of immunosuppression was observed in septic patients.

## Introduction

Sepsis is life-threatening organ dysfunction caused by a dysregulated host response to infection (Singer et al., 2016). As the most frequent cause of mortality in intensive care unit (ICU), sepsis is responsible for over 250,000 deaths each year in the United States (Martin, Mannino & Moss, 2006). Numerous studies have shown that the predominant driving force for morbidity and mortality of sepsis is immunosuppression (Hotchkiss, Monneret & Payen, 2013a; Hotchkiss & Nicholson, 2006; Hotchkiss & Opal, 2010; Hotchkiss et al., 2005; Sundar & Sires, 2013). Apoptosis of various immune cells has been proposed as a critical mediator of this sepsis-induced immunosuppression (Hotchkiss, Monneret & Payen, 2013b; Hotchkiss & Nicholson, 2006). Among these immune cells, a profound depletion of lymphocytes, including B cells, CD4^+^ and CD8^+^ T cells in different organs, has been reported (Hotchkiss, Monneret & Payen, 2013b; Hotchkiss & Nicholson, 2006). Different strategies to inhibit lymphocyte apoptosis have been proposed including anti-apoptotic cytokines and caspase inhibitors significantly improving sepsis prognosis (Hotchkiss et al., 2000; Oberholzer et al., 2001).

Extracellular histones, nuclear proteins released from injury or dying cells, are considered as major mediator of death in sepsis (Xu et al., 2009). During sepsis or major trauma, histones are released in the extracellular space and have been shown to contribute to the often fatal organ dysfunction (Kutcher et al., 2012; Xu et al., 2009). In our previous study, we demonstrated that extracellular histones induce lymphocyte apoptosis through mitochondria damage (Liu et al., 2013). However, the molecular events leading to mitochondria injury in lymphocytes treated with histones are not clear. Previous studies have shown that an increase in reactive oxygen species (ROS) production significantly alters mitochondrial functions and therefore represents a critical event in apoptosis (Galley, 2011; Victor et al., 2009). In sepsis, there is evidence that ROS production may be involved in lymphocyte apoptosis (Freeman et al., 2000; Galley, 2011; Victor et al., 2009). We therefore speculated that intracellular ROS levels may increase in lymphocytes treated with histones.

Proanthocyanidin, a class of polyphenols with strong antioxidant properties commonly found in a variety of plants, have been reported to exert cell protective effects by reducing mitochondria damage and inhibiting cell apoptosis (Gao et al., 2014; Lu et al., 2015; Zhen et al., 2014). Grape seed proanthocyanidin extract (GSPE), an extract from grape seed, contains phenolic hydroxyl groups, which are well-known antioxidants (Ding et al., 2013; Mansouri et al., 2011), safe and well tolerated in humans (Sano, in press). Antioxidants can scavenge free radicals and alleviate the peroxidation of membrane lipid, reducing free radical-related diseases and delaying aging (Lobo et al., 2010). Previous studies reported that GSPE exerts anti-inflammatory (Park et al., 2012; Zhou et al., 2011), anti-carcinogenic (Uchino et al., 2010), anti-mutagenic (Sharma & Katiyar, 2010), and anti-ischemia/reperfusion injury (Song et al., 2012; Wei et al., 2012) effects, as well as cardioprotective (Bagchi et al., 2003; Demirkaya et al., 2009) and neuroprotective (Ahn et al., 2011) benefits. Proanthocyanidin also contains catechin, a flavanol capable of crossing the blood–brain barrier more easily than other natural antioxidants (Zhen et al., 2014), such as quercetin (Shen et al., 2013) and curcumin (Ahmad, 2013). However, the role of GSPE on lymphocyte apoptosis via its anti-oxidative property is poorly defined. In the present study, we conducted in vitro experiments to assess the effect of GSPE on lymphocyte apoptosis caused by histones stimulation, and further explore its potential mechanism.

## Materials and Methods

### Chemicals and reagents

Grape seed proanthocyanidin extract was purchased from Tianjin Jianfeng Natural Product R&D Co., Ltd (Tianjin, China). Histones, *N*-acetylcysteine (NAC), Rhodamine123 and 2′,7′-dichlorofluorescin diacetate (DCFH-DA) were obtained from Sigma-Aldrich, Inc. (St. Louis, MO, USA). FITC Annexin V apoptosis detection kit was purchased from BD PharMingen (Franklin Lakes, NJ, USA).

### Subject

The study was conducted in accordance with the Declaration of Helsinki (2013), and the protocol was approved by the Ethics Committee of Zhujiang Hospital of Southern Medical University (2014-ZZYXK-004). All subjects gave their written informed consent to the work. Peripheral venous blood was drawn from 20- to 40-year-old healthy volunteers and was collected into vacuum tubes containing dried lithium heparin. Lymphocytes were isolated immediately after collection.

### Lymphocytes separation and stimulation

Lymphocyte separation medium (MP Biomedicals, Santa Ana, CA, USA) was used to isolate lymphocytes from freshly drawn blood according to the manufacturer’s instructions. Cells were cultured in RPMI 1640 medium (Gibco, Life Technology, Carlsbad, CA, USA) supplemented with 10% fetal bovine serum (FBS, Gibco, Australia origin) in a 5% CO_2_ incubator at 37 °C. Freshly isolated lymphocytes were added into a 12-well plate at a concentration of 1 × 10^6^ cells/ml and were divided into six or four groups: control (PBS), NAC, GSPE, histones, histones plus GSPE, and histones plus NAC. GSPE (2 μg/ml) and NAC (100 μM) were used to pre-treat lymphocytes for 2 h before stimulation with histones (50 μg/ml) for 2.5 h. Following histones stimulation, apoptosis, intracellular ROS levels, mitochondrial membrane potential, Bcl-2 expression, and caspase-3 cleavage were determined.

### Cellular assays

Lymphocyte apoptosis was determined by Annexin V and propidium iodide (PI) staining. Rhodamine 123 was used to measure mitochondrial membrane potential (Δψm) and DCFH-DA was used to detect intracellular ROS levels in lymphocytes. Annexin V/PI, Rho123, and DCFH-DA fluorescence was analyzed by flow cytometry (FACSCalibur, BD Biosciences, San Jose, CA, USA). Data were analyzed using FlowJo version 7.6 analysis software (Treestar, Inc., St. Ashland, OR, USA).

### Western blot analysis

RIPA buffer (Beyotime Biotechnology, Shanghai, China) was used to lyse the cells for total protein extraction. The BCA colorimetric assay (Beyotime Biotechnology, Shanghai, China) was used to quantify the amount of proteins in each sample, and 50 μg of protein were resolved by SDS-PAGE. Rabbit anti-human Bcl-2 (1:500 dilution, Cell Signaling Technology [CST], Danvers, MA, USA), rabbit anti-human cleaved caspase-3 (1:500 dilution, CST), and rabbit anti-human GAPDH (1:500 dilution, CST) were used as primary antibodies followed by HRP-conjugated goat anti-rabbit IgG antibodies (1:1,000 dilution, CST). The ECL reagent (Perkin-Elmer, Waltham, MA, USA) was used to detect immunolabeled proteins. Band intensities were quantified by the AlphaEase FC software (Alpha Innotech, San Leandro, CA, USA).

### Statistical analysis

Data were analyzed with GraphPad Prism version 5 (GraphPad software, Inc., San Diego, CA, USA). Results were expressed as mean ± SD. Two-way ANOVA was used to assess differences between groups and *P* < 0.05 was considered statistically significant.

## Results

### GSPE inhibited lymphocyte apoptosis induced by histones

**Figure 1:**
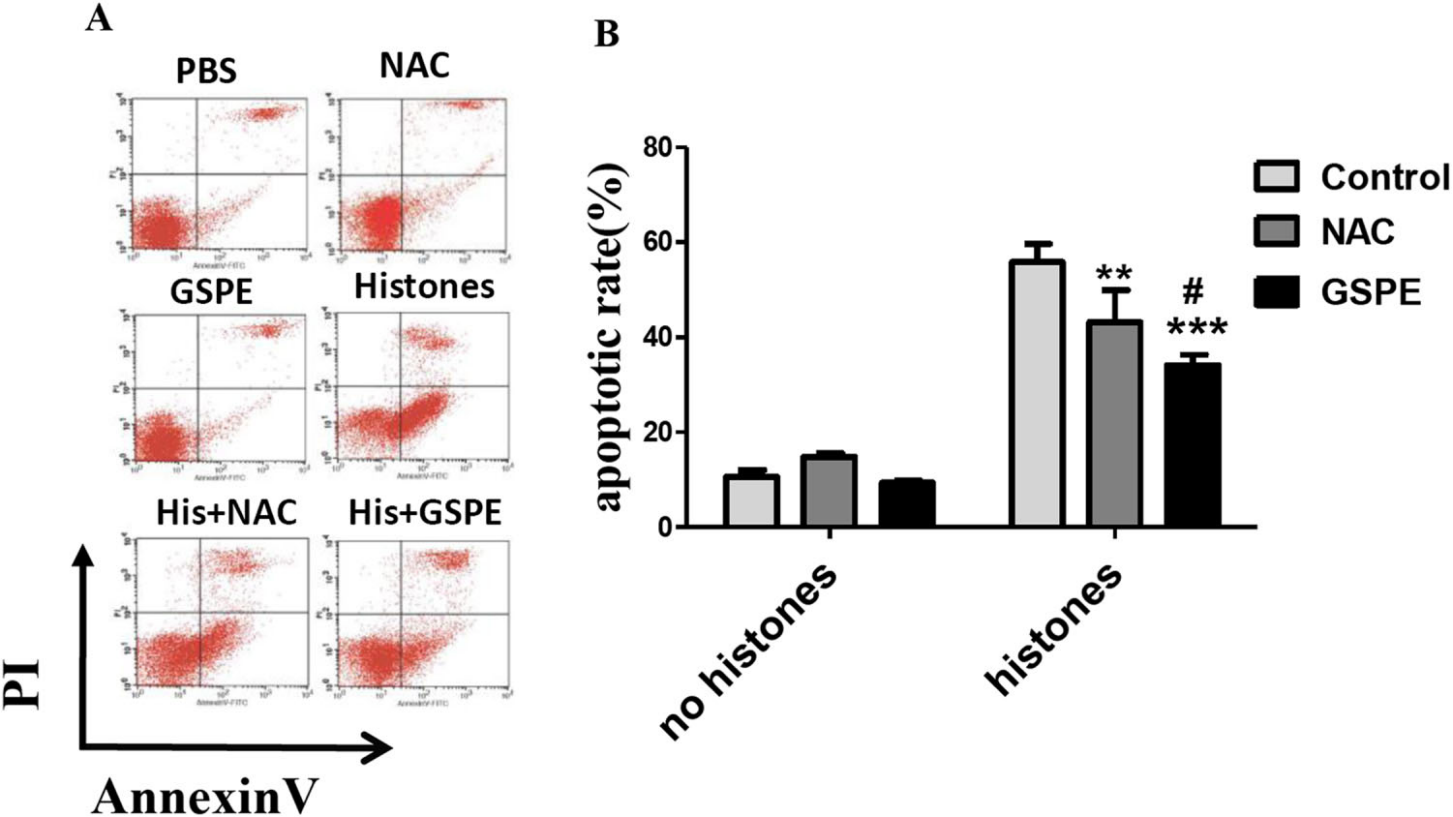
Grape seed proanthocyanidin extract inhibited lymphocyte apoptosis induced by histones. Human lymphocytes were cultured with PBS (control), GSPE (2 μg/ml), histones (His) (50 μg/ml), or histones (His) plus GSPE. NAC (100 μM) is used as a positive control of GSPE. Both GSPE and NAC were used to pre-treat cells for 2 h, then histones were added and cells cultured for an additional 2.5 h. Apoptosis were measured using Annexin V-FITC/PI double staining and flow cytometry analysis. (A) Representative pictures of lymphocytes apoptosis in indicated groups. Annexin V+ and PI− area represent early apoptosis, Annexin V+ and PI+ area represent late apoptosis. (B) Quantitative analysis of lymphocytes apoptosis. Total apoptotic lymphocytes (early and late apoptosis) were analyzed. There was a significant interaction between the effects of histones and GSPE on apoptosis, *F* = 15.76, Df = 2, *P* < 0.001. Df for error is 12. Values are presented as mean ±SD (*n* = 3). **P < 0.01, ***P < 0.001 vs control group treated with histones; #*P* < 0.05 vs NAC group treated with histones.

The concentration of extracellular histones increases in the serum of septic patients or mice resulting in lymphocyte damage (Liu et al., 2013). To test whether or not GSPE exerts protective effect on histone-induced lymphocyte apoptosis, we stimulated freshly isolated lymphocytes in vitro with histones in the presence or not of GSPE and assessed lymphocyte apoptosis by Annexin V/PI staining. As expected, the number of apoptotic cells increased significantly in histone-treated group compared with no histones control group (Fig. 1; 55.86 ± 3.00% vs 10.66 ± 1.44%, *P* < 0.001). Most of the apoptotic lymphocytes were found to be in the early stage of apoptosis (Annexin V+ and PI− cells). Furthermore, the time-dependent manner was also observed in histone stimulation (Fig. S1). GSPE significantly reduced lymphocyte apoptosis induced by histones (Fig. 1; 34.15 ± 2.15% vs 55.86 ± 3.00%, *P* = 0.001), particularly at the early stage of apoptosis. Similar effect was observed with the ROS inhibitor NAC (Fig. 1; 43.28 ± 6.64% vs 55.86 ± 3.00%, *P* < 0.05) and ascorbic acid (Fig. S2). A total of 1, 2, and 4 μg/ml GSPE all can reduce apoptosis, but 2 and 4 μg/ml concentrations have no significant difference (Fig. S3). So, 2 μg/ml GSPE was used in the followed experiments. GSPE at the concentration used in our experiments did not have significant toxicity on lymphocytes (Fig. 1). These results indicate that GSPE is capable of protecting lymphocytes against apoptosis induced by histones.

### GSPE-alleviated intracellular ROS formation caused by histone stimulation

**Figure 2:**
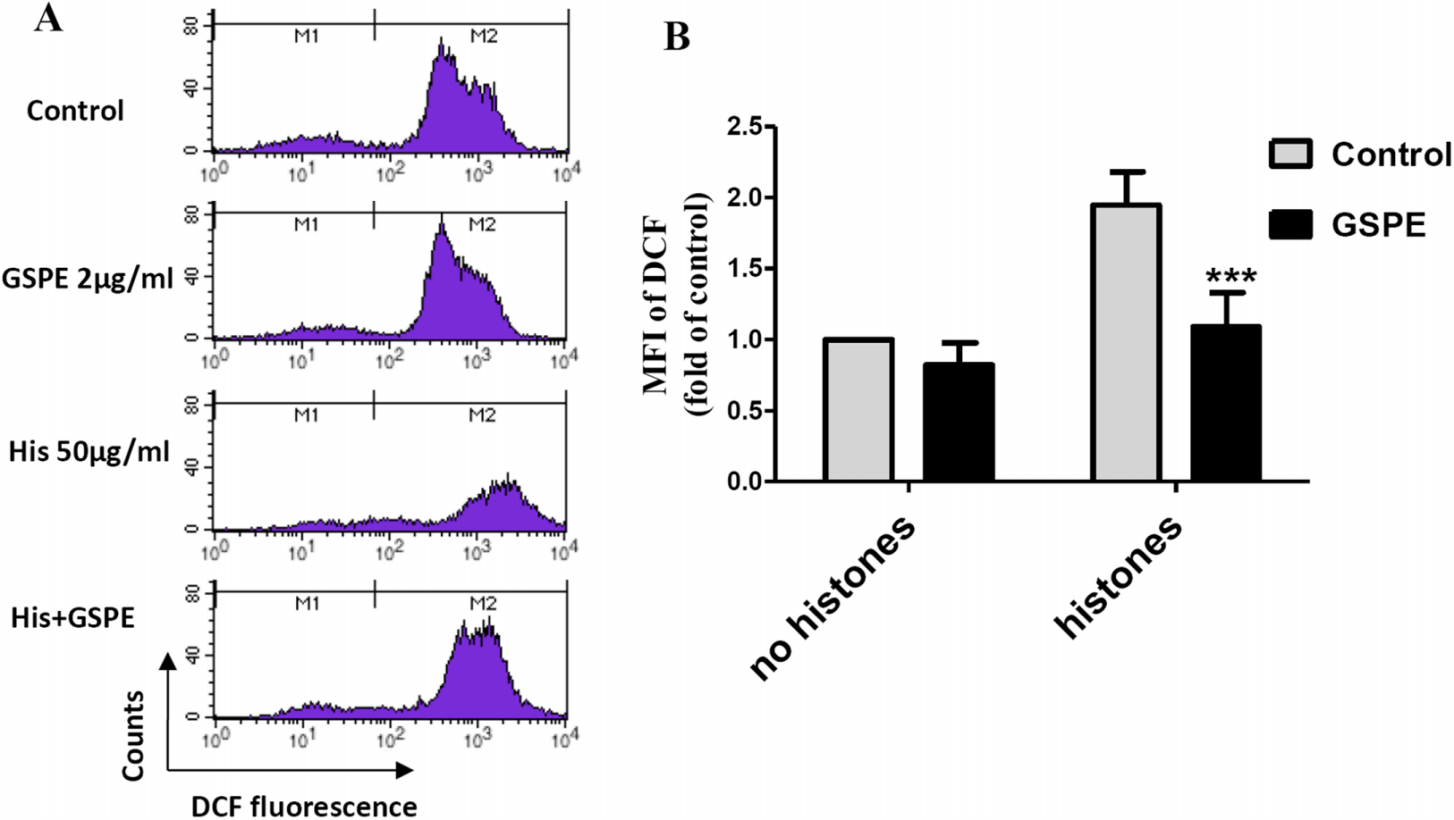
Grape seed proanthocyanidin extract decreased intracellular ROS formation caused by histones. Human lymphocytes were cultured with PBS (control), histones (His) (50 μg/ml), GSPE (2 μg/ml), or histones (His) plus GSPE. Cells were pre-treated with GSPE for 2 h, then histones were added and cells cultured for an additional 2.5 h. Intracellular ROS was measured by the fluorescent probe DCFH-DA and flow cytometry analysis. (A) Representative pictures of DCF-derived fluorescence in lymphocytes in indicated groups. M1 represents the ROS− cells, M2 represents the ROS+ cells. (B) Quantitative analysis of mean fluorescence intensity (MFI) of DCF. Results were expressed as the fold of control group without histones treatment. There was a significant interaction between the effects of histones and GSPE on ROS formation, *F* = 10.18, Df = 1, *P* < 0.05. Df for error is 8. Values are presented as mean ±SD (*n* = 3). ***P < 0.001 vs control group treated with histones.

Reactive oxygen species are well known to cause mitochondria membrane damage. To assess if the protective effect of GSPE on lymphocyte apoptosis is mediated by its antioxidant property, intracellular ROS levels were measured by DCFH-DA staining and flow cytometry analysis. A significant increase in intracellular ROS level was observed in histone-treated group vs no histones control group (Fig. 2; *P* < 0.01). Intracellular ROS levels were significantly reduced in the histones plus GSPE group compared with the histones group (Fig. 2; *P* < 0.01). No significant difference in intracellular ROS levels between GSPE and control groups without histones treatment were detected (Fig. 2). These data show that GSPE can inhibit intracellular ROS formation in lymphocyte treated with histones.

### GSPE prevented mitochondrial injury induced by histones

**Figure 3:**
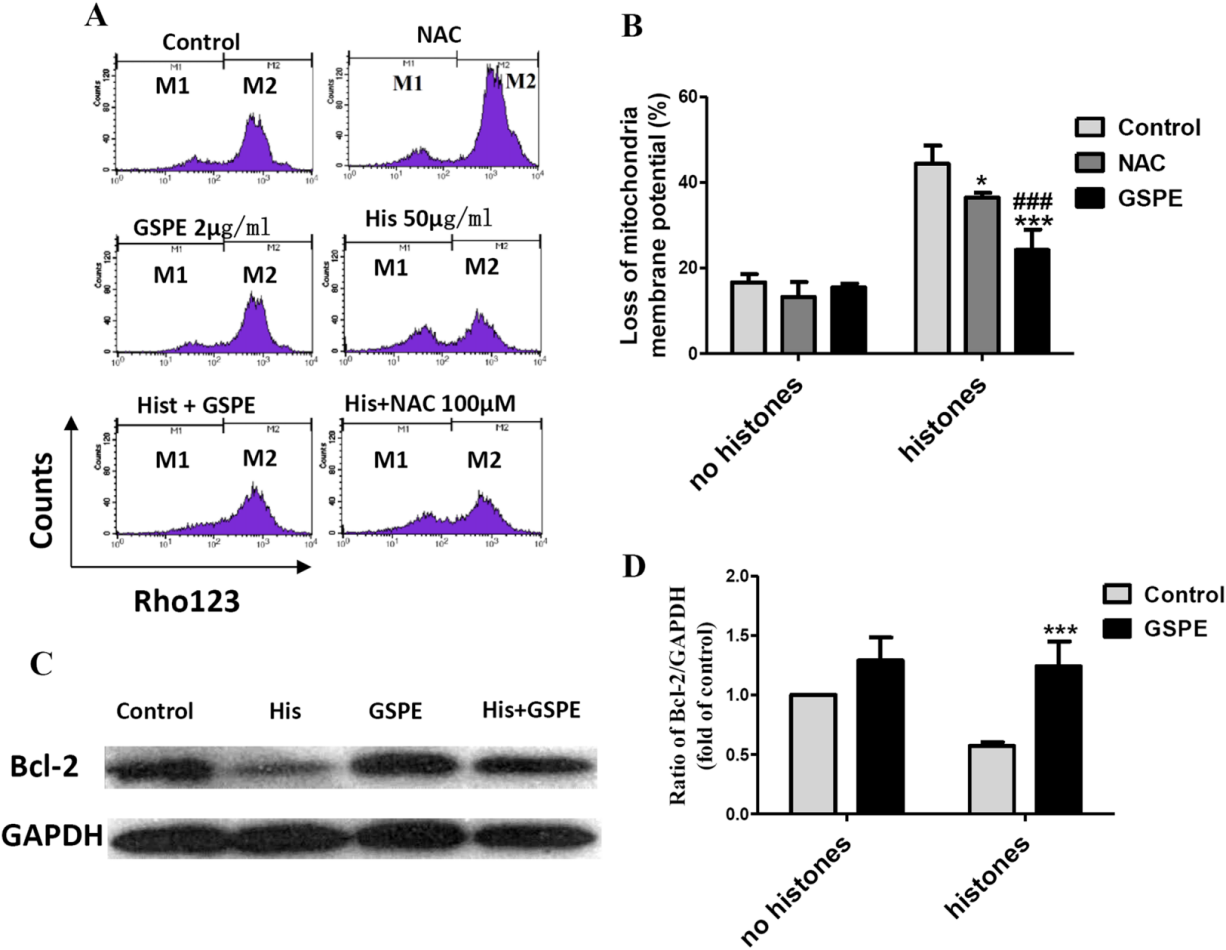
Grape seed proanthocyanidin extract inhibited mitochondrial damage caused by histones. Human lymphocytes were cultured with PBS (control), NAC (100 μM), histones (His) (50 μg/ml), GSPE (2 μg/ml), histones plus GSPE, or histones (His) plus NAC. Both GSPE and NAC were used to pre-treat cells for 2 h, then histones were added and cells cultured for an additional 2.5 h. Mitochondrial membrane potential (Δψm) was detected by Rhodamine (Rho) 123 and flow cytometry analysis. (A) Representative pictures of Rho 123 fluorescence in lymphocytes in indicated groups. M1 represents the percentage of lymphocytes with Δψm loss, M2 represents the percentage of lymphocytes without Δψm loss. (B) Bar graph showing differences in the percentage of cells with Δψm loss. There was a significant interaction between the effects of histones and GSPE on Δψm loss, *F* = 15.31, Df = 1, *P* < 0.001. Df for error is 12. Values were presented as mean ±SD (*n* = 3). *P < 0.05, ***P < 0.001 vs control group treated with histones; ###*P* < 0.001 vs NAC group treated with histones. (C) Representative blots of Bcl-2 expression in lymphocytes in indicated groups. Human lymphocytes were cultured with PBS (control), histone (His) (50 μg/ml), GSPE (2 μg/ml), or histones (His) plus GSPE. GSPE was used to pre-treat cells for 2 h, then histones were added and cultured for 2.5 h. Bcl-2 expression was evaluated by western blotting. GAPDH was used as a loading control to normalize data for differences in the amount of total proteins loaded per lane. (D) Quantification of Bcl-2/GAPDH expression ratio. Densitometric analysis of relative bcl-2 band intensities were used to compare groups. Results were expressed as fold change compared to control group without histones treatment. The main effects of histones and GSPE are significant. For histones, *F* = 8.22, Df = 1, *P* < 0.05, for GSPE, *F* = 33.12, Df = 1, *P* < 0.001. Df for error is 8. Values are presented as mean ±SD (*n* = 3). ***P < 0.001 vs control group treated with histones.

Mitochondrial injury is a key event in cell apoptosis. In order to determine whether GSPE can protect lymphocyte apoptosis by limiting mitochondrial injury, the mitochondria membrane potential (Δψm), an indicator of mitochondrial injury, was measured using Rhodamine 123 (Rho123) staining followed by flow cytometry analysis. The loss of lymphocyte Δψm observed in the histone-treated group compared to control group (as indicated by the increase in the number of events in the M1 gate) was significantly reduced by GSPE treatment (Figs. 3A and 3B). NAC treatment also alleviated the loss of lymphocyte Δψm induced by histones, but was less potent than GSPE in reducing mitochondrial damage caused by histones (Figs. 3A and 3B).

Bcl-2 is an anti-apoptotic regulator involved in preventing the release of apoptogenic factors located in the mitochondria. Histones treatment resulted in a substantial diminution of Bcl-2 expression in lymphocytes (Figs. 3C and 3D, *P* < 0.05). Pretreatment of lymphocytes with GSPE was able to counteract the downregulation of Bcl-2 expression induced by histones (Figs. 3C and 3D, *P* < 0.01).

The opening of mitochondrial permeability transition (MPT) pore can reduce Δψm and disturb the mitochondrial membrane stability. In our previous report, cyclosporin A (CsA), an inhibitor of mitochondrial permeability transition, was identified to protect lymphocytes from apoptosis induced by histone stimulation (Liu et al., 2013). Therefore, the effect of GSPE in preventing mitochondrial membrane damage caused by histones is consistent with our previous observation using CsA. Altogether these data suggest that GSPE has the ability to protect mitochondria of lymphocytes treatment with histones.

### GSPE blocked caspase-3 activation induced by histones

**Figure 4:**
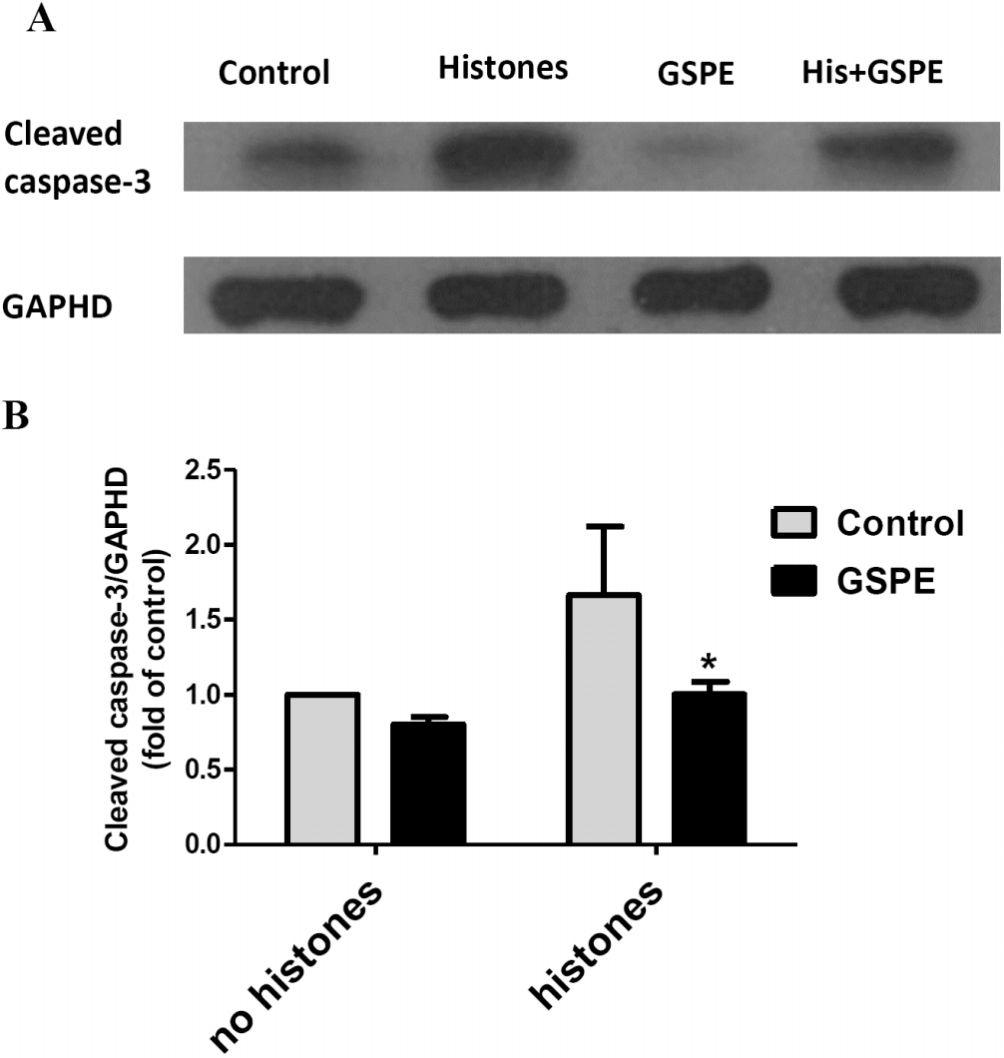
Grape seed proanthocyanidin extract inhibited caspase-3 activation induced by histones. Human lymphocytes were cultured with PBS (control), histones (His) (50 μg/ml), GSPE (2 μg/ml), or histones (His) plus GSPE. GSPE was used to pre-treat cells for 2 h, then histones were added and cells cultured for an additional 2.5 h. Control cells were treated with PBS. Cleaved caspase-3 were detected by western blotting. GAPDH was used as a loading control to normalize data for differences in the amount of total proteins loaded per lane. (A) Representative blots of cleaved caspase-3 expression for each group are shown. Cleaved caspase-3 represents the activated form of caspase-3. (B) Quantification of cleaved caspase-3/GAPDH expression ratio. Densitometric analysis of relative cleaved caspase-3 band intensities were used to compare groups. Results were expressed as the fold change compared to control group without histones treatment. The main effects of histones and GSPE are significant. For histones, *F* = 10.48, Df = 1, *P* < 0.05, for GSPE, *F* = 10.32, Df = 1, *P* < 0.05. Df for error is 8. Values are presented as mean ±SD (*n* = 3). *P < 0.05 vs control group treated with histones.

Apoptosis mediated by both intrinsic (mitochondria) and extrinsic (death receptors) pathways eventually leads to caspase-3 activation (cleavage). To further understand the protective effect of GSPE on lymphocyte apoptosis, we measured caspase-3 activation by western-blot analysis. Relative density analysis of cleaved caspase-3 protein bands was used to compare the activation of caspase-3 between groups. Cleavage of caspase-3 was strongly upregulated by histones treatment (Fig. 4; *P* < 0.05) and this effect of histones on caspase-3 activation was significantly reduced by GSPE treatment (Fig. 4; *P* < 0.05).

## Discussion

During sepsis, lymphocyte apoptosis severely aggravates immunosuppression, and as a consequence negatively impacts disease prognosis (Hotchkiss & Karl, 2003; Hotchkiss, Monneret & Payen, 2013a; Hotchkiss et al., 1999, 2005). Our previous report, using a mouse model of sepsis, demonstrated that histones, nuclear proteins released from dying cells, mediated lymphocytes apoptosis by inducing mitochondrial damage (Liu et al., 2013). In this study, we found that GSPE pre-treatment significantly suppressed histones-mediated lymphocyte apoptosis. ROS generation and mitochondrial damage caused by histones stimulation were inhibited by GSPE pre-treatment.

Apoptosis is mediated by three main cellular pathways: the ER stress-induced pathway, the extrinsic death receptor pathway, and the intrinsic/mitochondrial pathway (Kwak, 2013). Cleavage of caspase-3, a primary executioner of apoptosis, is the ultimate downstream event common to all three pathways (Kwak, 2013), and is therefore considered as a marker of cell apoptosis. In the present study, we showed that GSPE treatment significantly reduced the cleavage of caspase-3 induced by histones stimulation which is consistent with the anti-apoptotic effect of GSPE previously observed in the perfluorooctanoic acid-induced hepatotoxicity model (Liu et al., 2016). In addition, several other natural antioxidants, such as *Bauhinia championii* flavone, cyanidin-3-glucoside, and *N*-acetyl cysteine (NAC) have been previously reported to exert anti-apoptotic effects in cardiomyocytes (Jian et al., 2016), neurons (Ke et al., 2011), and human lymphocytes (Han et al., 2004), respectively.

Mitochondria damage is a key event in cell apoptosis (Kwak, 2013). High expression of Bcl-2, an anti-apoptotic protein residing in the outer mitochondrial membrane, helps to maintain mitochondrial membrane integrity (Kwak, 2013). A reduction in Bcl-2 expression leads to a loss of mitochondrial membrane potential and the permeabilization of the mitochondrial outer membrane, which results in the release of the pro-apoptotic factor cytochrome c and activation of caspase-3 (Kwak, 2013). Our results showed that GSPE reduces the Δψm loss as well as the decrease in Bcl-2 expression caused by histones stimulation. In accordance with our results, previous studies have shown that the natural antioxidants, *B. championii* flavone and NAC also protect mitochondria by reducing the loss of Δψm or by enhancing Bcl-2 expression (Han et al., 2004; Jian et al., 2016). In addition, cyclosporine (CsA), an inhibitor of MPT, also reduces lymphocyte apoptosis induced by histones, as described in our previous report (Liu et al., 2013). Therefore, our data suggest that GSPE protects lymphocytes from apoptosis induced by histones maybe related with its effect on Mitochondria.

Reactive oxygen species, a byproduct of oxygen metabolism, increase dramatically during environmental stress and induce cellular damage, which often leads to apoptosis (Sun et al., 2015). Mitochondria are one of the main targets affected by intracellular ROS accumulation. Indeed, intracellular ROS accumulation provokes mitochondrial membrane instability, pro-apoptotic cytochrome c release and finally caspase-3 activation (Yu et al., 2016). Our results show for the first time that histones mediate a significant accumulation of intracellular ROS in lymphocytes. Similarly, the high-mobility group box 1 (HMGB1), another nuclear protein released by necrotic cells, was previously shown to induce ROS production in a liver ischemia/reperfusion (I/R) model (Tsung et al., 2007). Treatment of lymphocytes with GSPE prior to histones stimulation significantly reduced the intracellular accumulation of ROS. Interestingly, NAC, a strong ROS scavenging agent was also capable to significantly decrease mitochondrial damage and lymphocyte apoptosis but less efficiently than GSPE. Accumulation of ROS has been shown to induce vascular endothelium damage in sepsis (Cepinskas & Wilson, 2008). Our data showed that GSPE treatment may be beneficial in sepsis because of the ability to reduce ROS formation.

Altogether our data indicate that ROS accumulation is involved in histones-induced apoptosis of lymphocytes. We also show that GSPE, a natural antioxidant, can protect lymphocytes from apoptotic cell death. Also, the inhibition of intracellular ROS formation and mitochondria injury were observed in this study. Because lymphocyte apoptosis has been shown to be a critical event in the systemic immunosuppression observed in septic patients, these findings could contribute to the development of novel sepsis therapies. Further experiments are necessary to evaluate the effect of GSPE on the other apoptotic pathways.

## Supplemental Information

10.7717/peerj.3108/supp-1 Supplemental Information 1 Raw data for Apoptosis.

10.7717/peerj.3108/supp-2 Supplemental Information 2 Raw data for Apoptosis.

10.7717/peerj.3108/supp-3 Supplemental Information 3 Raw data for Apoptosis.

10.7717/peerj.3108/supp-4 Supplemental Information 4 Bcl-2: Control, Histones, GSPE, Histones + GSPE.

10.7717/peerj.3108/supp-5 Supplemental Information 5 Cleaved Caspase-3: Control, Histones, GSPE, Histones + GSPE.

10.7717/peerj.3108/supp-6 Supplemental Information 6 Histones induced lymphocyte apoptosis in a time dependent manner. Human lymphocytes were cultured with histones(50 μg/ml) for 0, 0.5, 1 and 2.5 h respectively. Apoptosis were measured using AnnexinV-FITC/PI double staining and flow cytometry analysis. Quantitative analysis of lymphocytes apoptosis was performed. Total apoptotic lymphocytes (early and late apoptosis) were analyzed. Values are presented as means ±SD (*n* = 3). ***P < 0.001 vs 0 h; ^&&^*P* < 0.01, ^&^*P* < 0.05 vs 0.5 h.

10.7717/peerj.3108/supp-7 Supplemental Information 7 Ascorbic acid inhibits histones-induced lymphocyte apoptosis. Human lymphocytes were cultured with PBS (control), ascorbic acid (AA) (25 μM), histones (His) (50 μg/ml) and histones (His) plus AA. AA was used to pre-treat cells for 6 h, then histones were added and cells cultured for an additional 2.5 h. Apoptosis were measured using AnnexinV-FITC/PI double staining and flow cytometry analysis. (A) Representative pictures of lymphocytes apoptosis in indicated groups. Annexin V+ and PI− area represent early apoptosis, Annexin V+ and PI+ area represent late apoptosis. (B) Quantitative analysis of lymphocytes apoptosis. Total apoptotic lymphocytes (early and late apoptosis) were analyzed. There was a significant interaction between the effects of histones and AA on apoptosis, *F* = 12.31, Df = 1, *P* < 0.01. Values are presented as means ±SD (*n* = 3). **P < 0.01 vs control group.

10.7717/peerj.3108/supp-8 Supplemental Information 8 GSPE inhibits histones-induced lymphocyte apoptosis in a dose dependent manner. Human lymphocytes were cultured with PBS, histones (His) (50 μg/ml), His+GSPE (1 μg/ml), His+GSPE (2 μg/ml), His+GSPE (4 μg/ml). GSPE was used to pre-treat cells for 2 h, then histones were added and cells cultured for an additional 2.5 h. Apoptosis were measured using AnnexinV-FITC/PI double staining and flow cytometry analysis. Quantitative analysis of lymphocytes apoptosis was performed. Total apoptotic lymphocytes (early and late apoptosis) were analyzed. Values are presented as means ±SD (*n* = 3). ***P < 0.001, **P < 0.01, *P < 0.05 vs PBS; ^&&&^*P* < 0.001 vs histones (50 μg/ml); #*P* < 0.05 vs His+GSPE (1 μg/ml).

## Additional Information and Declarations

### Data Deposition

The following information was supplied regarding data availability: The raw data has been supplied as Supplemental Dataset Files.
