# Supplementary material for: Grape seed proanthocyanidin extract protects lymphocytes against histone-induced apoptosis
Source: PeerJ. 2017 Mar 21;5:e3108. doi: 10.7717/peerj.3108 (PMC5363264; doi:10.7717/peerj.3108)
Supplement: Supplemental Information 1 [file peerj-05-3108-s001.pdf]

Raw data of apoptosis

Control

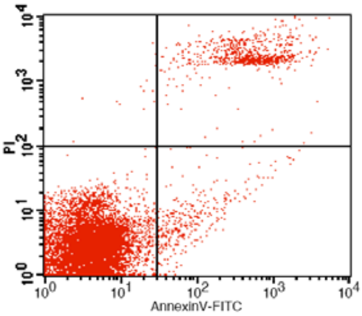

Quad	Events	% Gated	% Total
UL	14	0.14	0.06
UR	789	7.89	3.44
LL	8882	88.82	38.68
LR	315	3.15	1.37

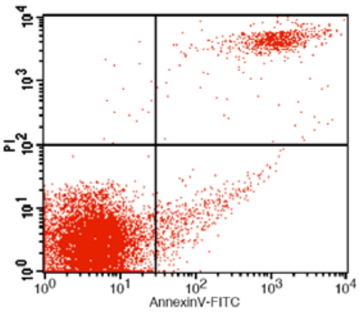

Quad	Events	% Gated	% Total
UL	15	0.15	0.07
UR	737	7.37	3.26
LL	8798	87.98	38.94
LR	450	4.50	1.99

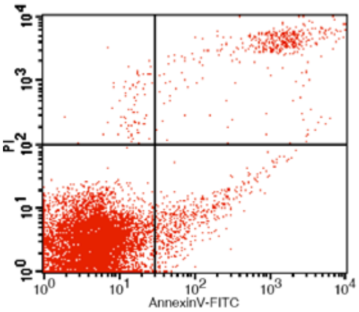

Quad	Events	% Gated	% Total
UL	78	0.78	0.34
UR	419	4.19	1.81
LL	9016	90.16	39.05
LR	487	4.87	2.11

GSPE

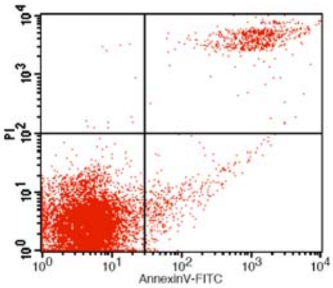

Quad	Events	% Gated	% Total
UL	13	0.13	0.06
UR	675	6.75	3.09
LL	8936	89.36	40.97
LR	376	3.76	1.72

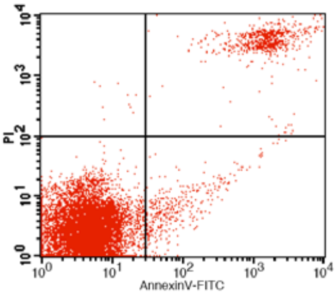

Quad	Events	% Gated	% Total
UL	10	0.10	0.05
UR	687	6.87	3.32
LL	8952	89.52	43.20
LR	351	3.51	1.69

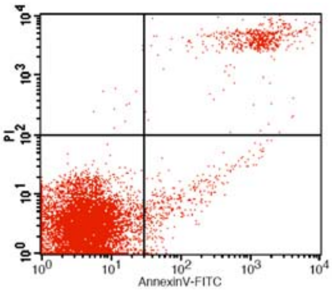

Quad	Events	% Gated	% Total
UL	11	0.11	0.05
UR	576	5.76	2.71
LL	9063	90.63	42.72
LR	350	3.50	1.65

Histones

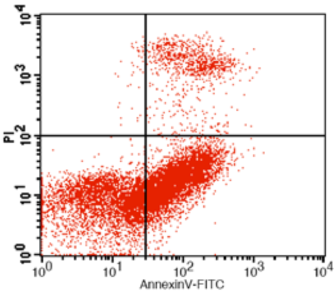

Quad	Events	% Gated	% Total
UL	45	0.45	0.07
UR	912	9.12	1.37
LL	3964	39.64	5.94
LR	5079	50.79	7.62

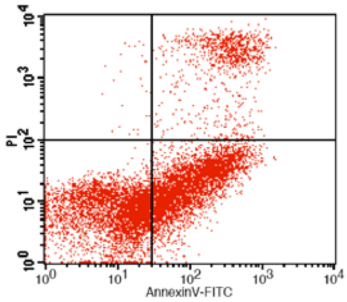

Quad	Events	% Gated	% Total
UL	28	0.28	0.06
UR	1014	10.14	2.16
LL	4447	44.47	9.48
LR	4511	45.11	9.62

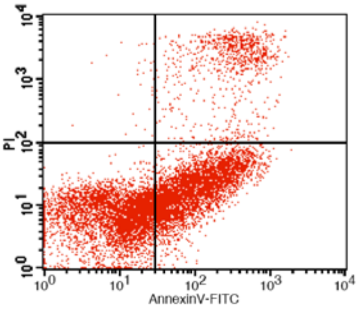

Quad	Events	% Gated	% Total
UL	32	0.32	0.07
UR	821	8.21	1.76
LL	4725	47.25	10.12
LR	4422	44.22	9.47

Histones + GSPE

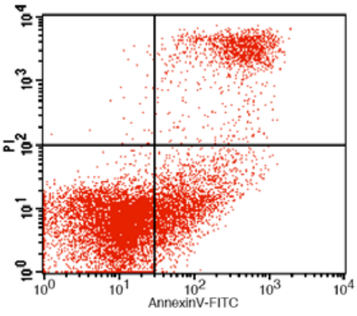

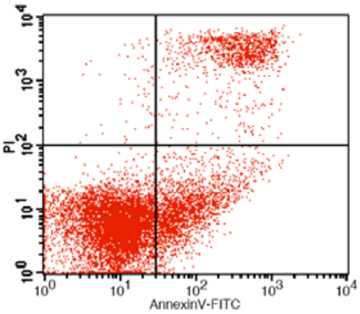

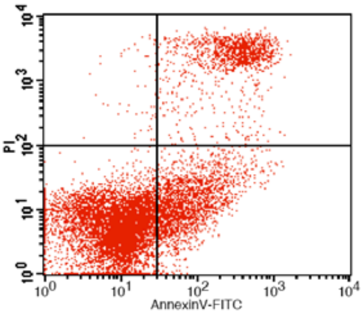

Quad	Events	% Gated	% Total
UL	33	0.33	0.08
UR	1358	13.58	3.16
LL	6376	63.76	14.85
LR	2233	22.33	5.20

Quad	Events	% Gated	% Total
UL	59	0.59	0.16
UR	1129	11.29	2.99
LL	6765	67.65	17.89
LR	2047	20.47	5.41

Quad	Events	% Gated	% Total
UL	56	0.56	0.12
UR	1306	13.06	2.84
LL	6466	64.66	14.05
LR	2172	21.72	4.72

Histones + NAC

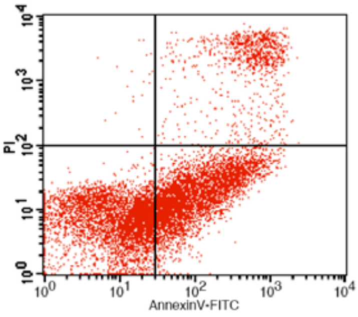

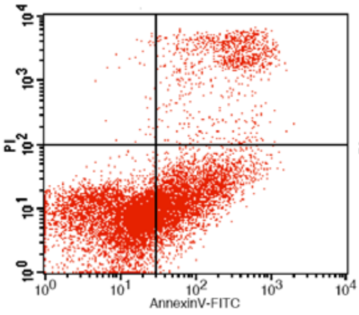

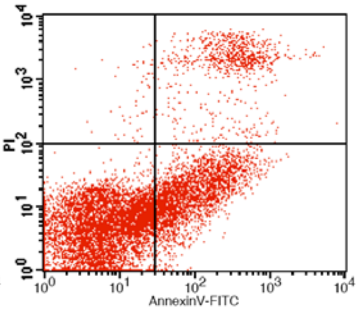

Quad	Events	% Gated	% Total
UL	21	0.21	0.04
UR	903	9.03	1.92
LL	4529	45.29	9.62
LR	4547	45.47	9.65

Quad	Events	% Gated	% Total
UL	32	0.32	0.07
UR	970	9.70	2.22
LL	5433	54.33	12.43
LR	3565	35.65	8.16

Quad	Events	% Gated	% Total
UL	54	0.54	0.14
UR	832	8.32	2.14
LL	6236	62.36	16.03
LR	2878	28.78	7.40
