# Supplementary figures and images for: Grape seed proanthocyanidin extract protects lymphocytes against histone-induced apoptosis
Source: PeerJ. 2017 Mar 21;5:e3108. doi: 10.7717/peerj.3108 (PMC5363264; doi:10.7717/peerj.3108)

# Raw data of apoptosis

## Control

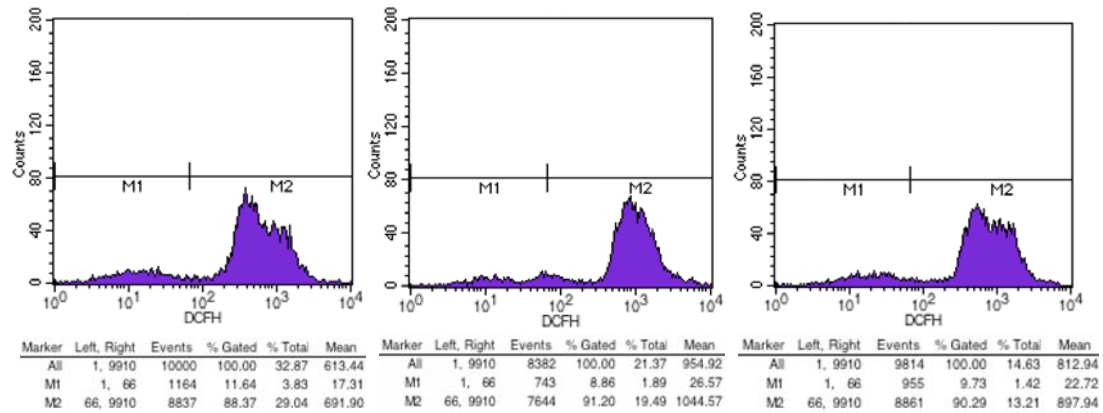

## GSPE

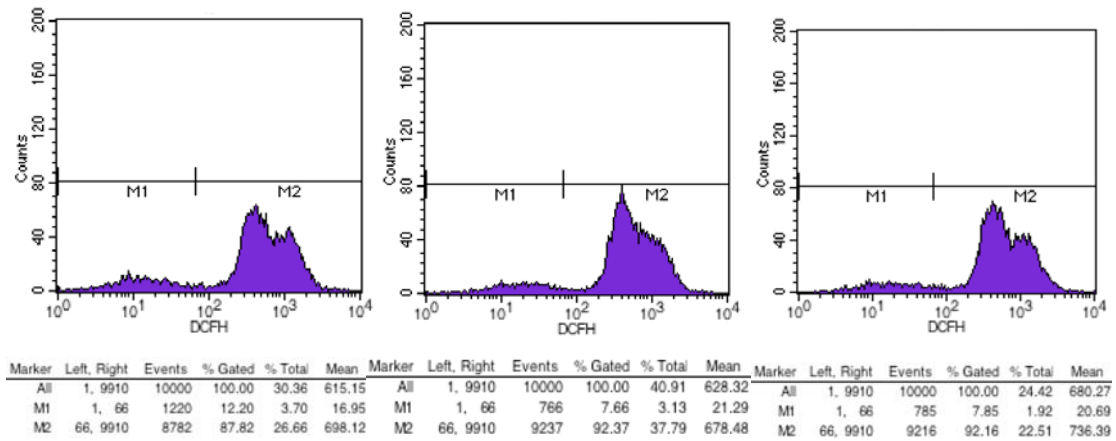

## Histones

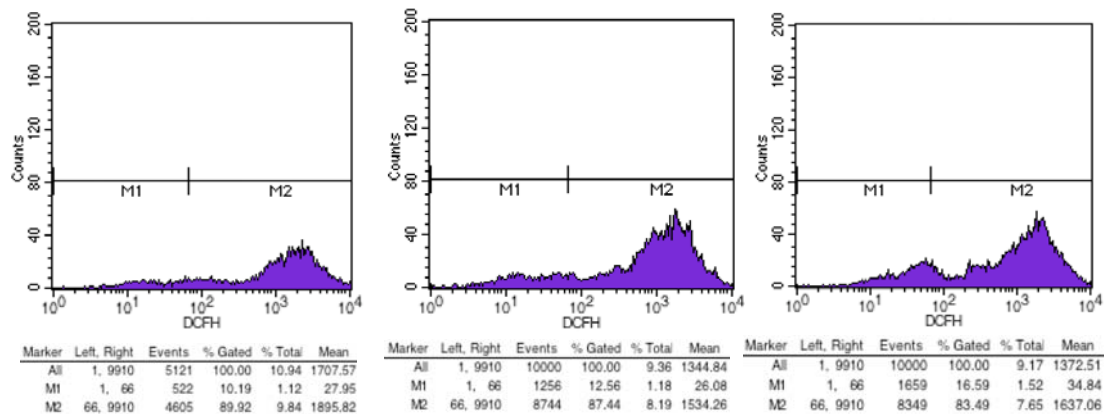

Histones + GSPE

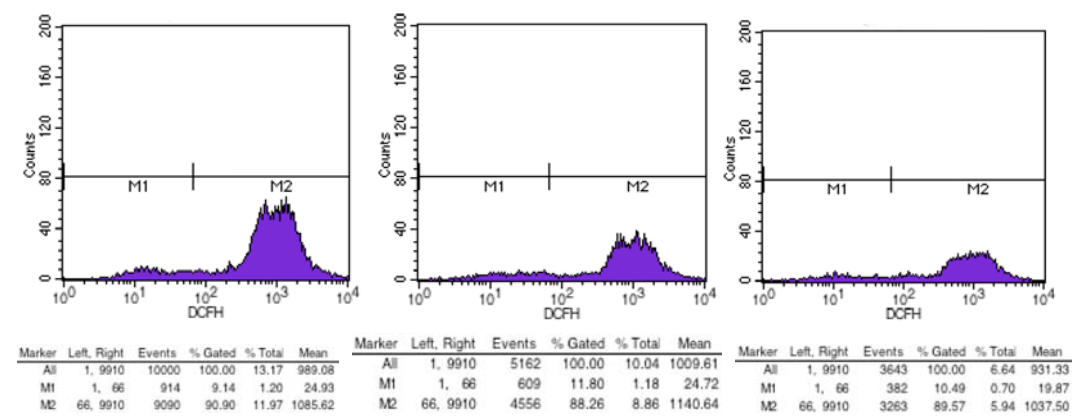

Supplement: Supplemental Information 2 [file peerj-05-3108-s002.pdf]

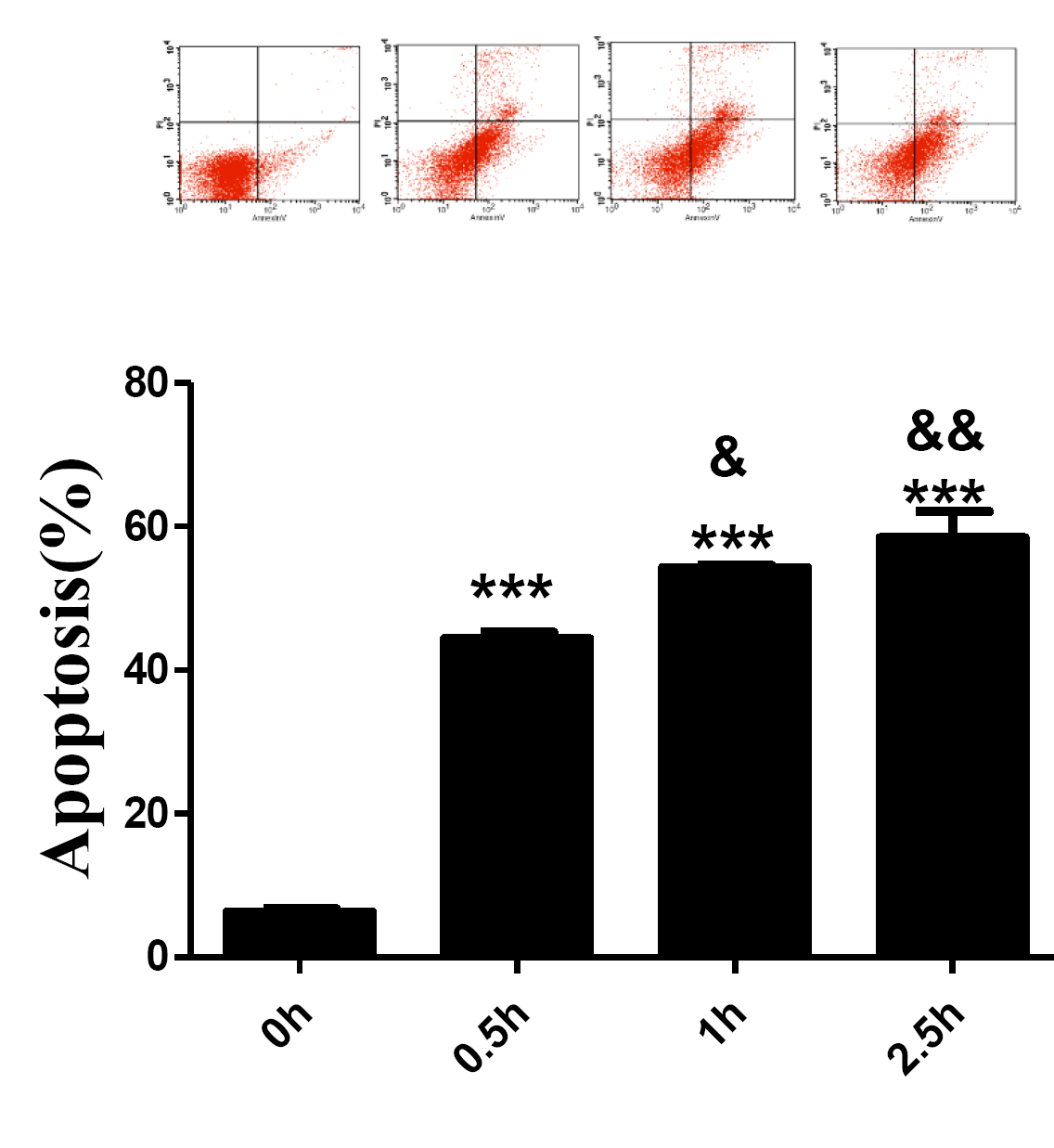

Supplement: Supplemental Information 6 — Human lymphocytes were cultured with histones(50 μg/ml) for 0, 0.5, 1 and 2.5 h respectively. Apoptosis were measured using AnnexinV-FITC/PI double staining and flow cytometry analysis. Quantitative analysis of lymphocytes apoptosis was performed. Total apoptotic lymphocytes (early and late apoptosis) were analyzed. Values are presented as means ±SD (n = 3). ***P < 0.001 vs 0 h; &&P < 0.01, &P < 0.05 vs 0.5 h. [file peerj-05-3108-s006.png]

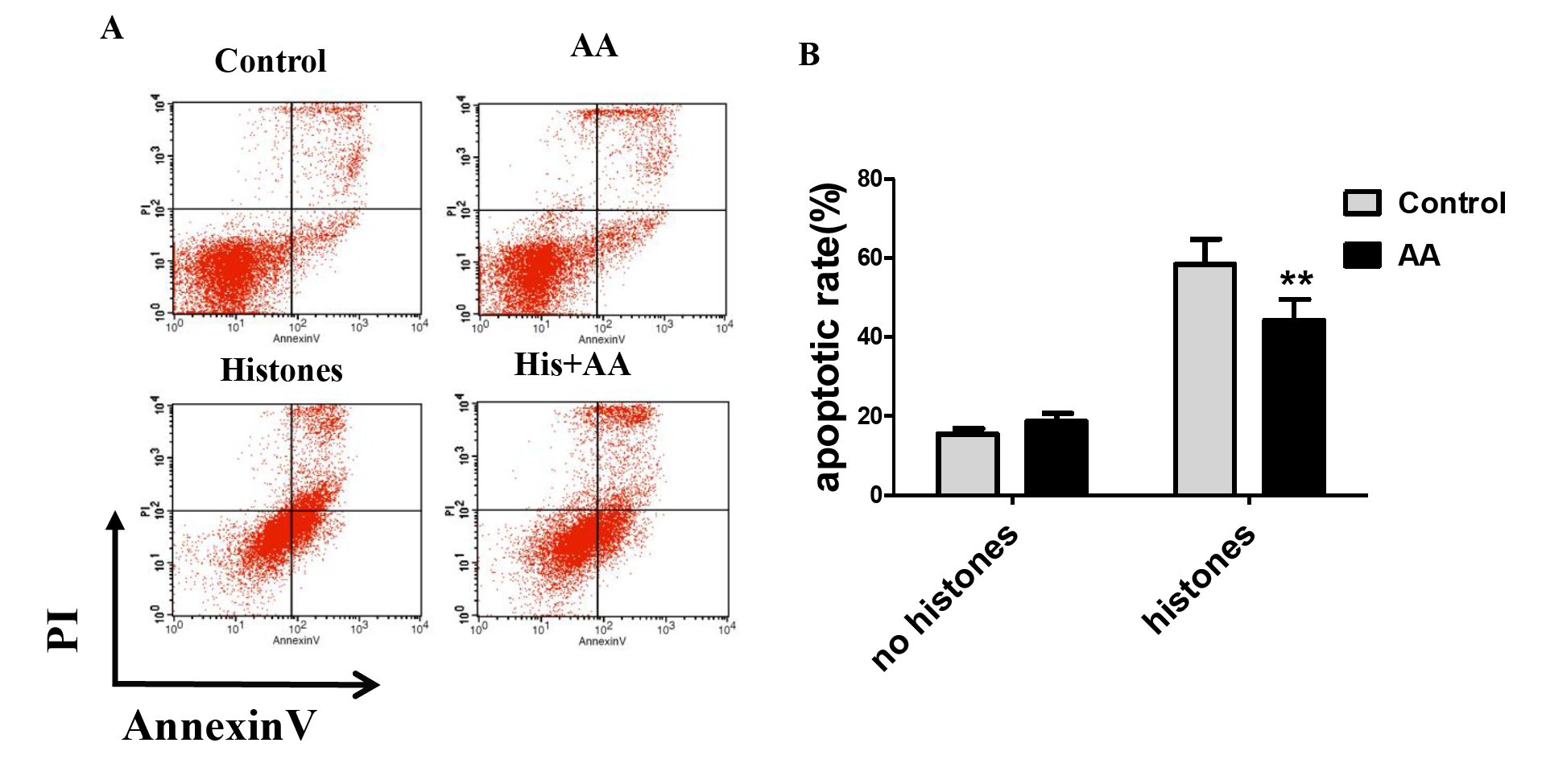

Supplement: Supplemental Information 7 — Human lymphocytes were cultured with PBS (control), ascorbic acid (AA) (25 μM), histones (His) (50 μg/ml) and histones (His) plus AA. AA was used to pre-treat cells for 6 h, then histones were added and cells cultured for an additional 2.5 h. Apoptosis were measured using AnnexinV-FITC/PI double staining and flow cytometry analysis. (A) Representative pictures of lymphocytes apoptosis in indicated groups. Annexin V+ and PI− area represent early apoptosis, Annexin V+ and PI+ area represent late apoptosis. (B) Quantitative analysis of lymphocytes apoptosis. Total apoptotic lymphocytes (early and late apoptosis) were analyzed. There was a significant interaction between the effects of histones and AA on apoptosis, F = 12.31, Df = 1, P < 0.01. Values are presented as means ±SD (n = 3). **P < 0.01 vs control group. [file peerj-05-3108-s007.png]

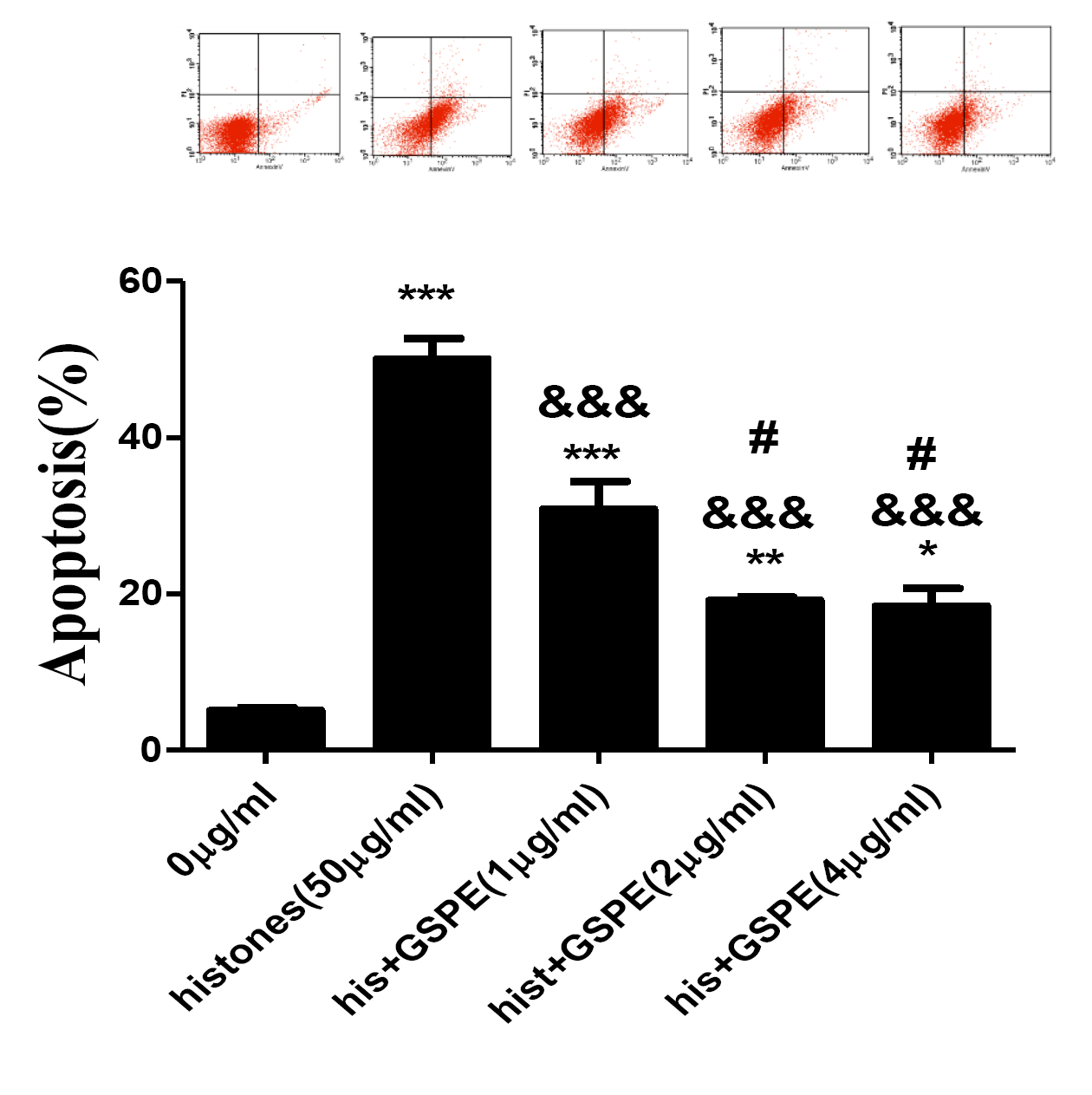

Supplement: Supplemental Information 8 — Human lymphocytes were cultured with PBS, histones (His) (50 μg/ml), His+GSPE (1 μg/ml), His+GSPE (2 μg/ml), His+GSPE (4 μg/ml). GSPE was used to pre-treat cells for 2 h, then histones were added and cells cultured for an additional 2.5 h. Apoptosis were measured using AnnexinV-FITC/PI double staining and flow cytometry analysis. Quantitative analysis of lymphocytes apoptosis was performed. Total apoptotic lymphocytes (early and late apoptosis) were analyzed. Values are presented as means ±SD (n = 3). ***P < 0.001, **P < 0.01, *P < 0.05 vs PBS; &&&P < 0.001 vs histones (50 μg/ml); #P < 0.05 vs His+GSPE (1 μg/ml). [file peerj-05-3108-s008.png]
