# Supplementary material for: Grape seed proanthocyanidin extract protects lymphocytes against histone-induced apoptosis
Source: PeerJ. 2017 Mar 21;5:e3108. doi: 10.7717/peerj.3108 (PMC5363264; doi:10.7717/peerj.3108)
Supplement: Supplemental Information 3 [file peerj-05-3108-s003.pdf]

Raw data of apoptosis

Control

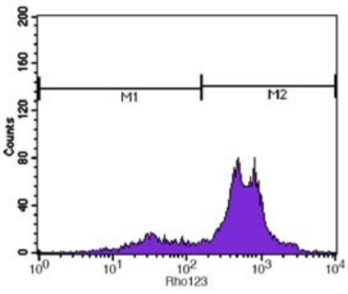

Marker	Left, Right	Events	% Gated	% Total
All	1, 9910	10000	100.00	62.29
M1	1, 155	1779	17.79	11.08
M2	155, 9910	8228	82.28	51.25

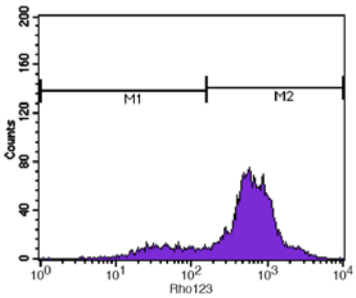

Marker	Left, Right	Events	% Gated	% Total
All	1, 9910	10000	100.00	66.19
M1	1, 155	1447	14.47	9.58
M2	155, 9910	8560	85.60	56.65

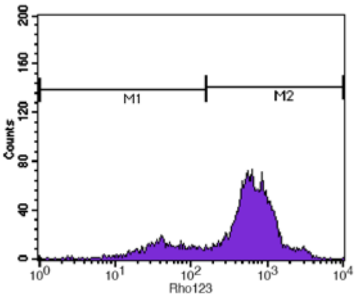

Marker	Left, Right	Events	% Gated	% Total
All	1, 9910	10000	100.00	59.60
M1	1, 155	1781	17.81	10.62
M2	155, 9910	8224	82.24	49.02

GSPE

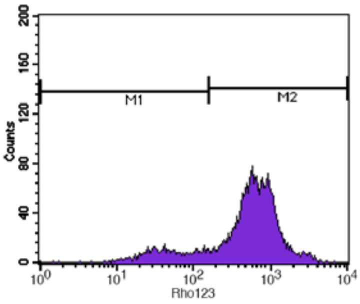

Marker	Left, Right	Events	% Gated	% Total
All	1, 9910	10000	100.00	64.38
M1	1, 155	1463	14.63	9.42
M2	155, 9910	8542	85.42	54.99

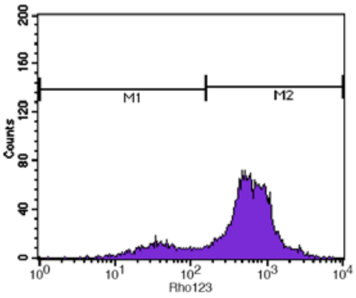

Marker	Left, Right	Events	% Gated	% Total
All	1, 9910	10000	100.00	63.17
M1	1, 155	1577	15.77	9.96
M2	155, 9910	8432	84.32	53.27

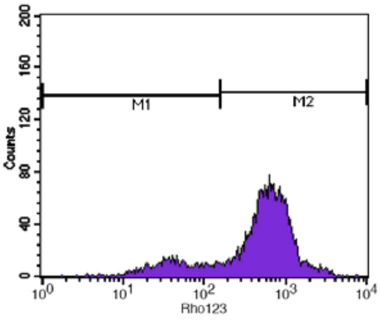

Marker	Left, Right	Events	% Gated	% Total
All	1, 9910	10000	100.00	59.59
M1	1, 155	1627	16.27	9.69
M2	155, 9910	8382	83.82	49.95

Histones

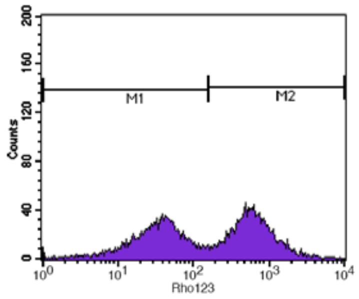

Marker	Left, Right	Events	% Gated	% Total
All	1, 9910	10000	100.00	20.68
M1	1, 155	4927	49.27	10.19
M2	155, 9910	5079	50.79	10.50

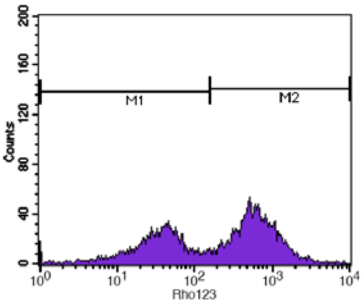

Marker	Left, Right	Events	% Gated	% Total
All	1, 9910	10000	100.00	30.08
M1	1, 155	4177	41.77	12.56
M2	155, 9910	5830	58.30	17.53

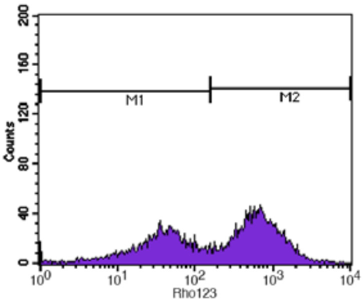

Marker	Left, Right	Events	% Gated	% Total
All	1, 9910	10000	100.00	30.48
M1	1, 155	4216	42.16	12.85
M2	155, 9910	5790	57.90	17.65

Histones + GSPE

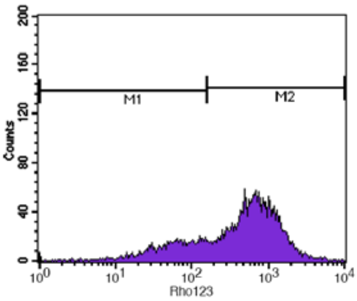

Marker	Left, Right	Events	% Gated	% Total
All	1, 9910	10000	100.00	27.74
M1	1, 155	2360	23.60	6.55
M2	155, 9910	7655	76.55	21.23

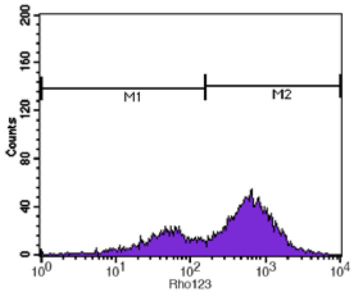

Marker	Left, Right	Events	% Gated	% Total
All	1, 9910	10000	100.00	30.28
M1	1, 155	2932	29.32	8.88
M2	155, 9910	7074	70.74	21.42

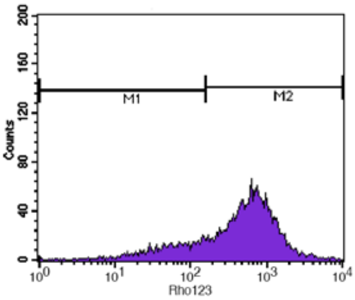

Marker	Left, Right	Events	% Gated	% Total
All	1, 9910	10000	100.00	27.93
M1	1, 155	1991	19.91	5.56
M2	155, 9910	8025	80.25	22.41

Histones + NAC

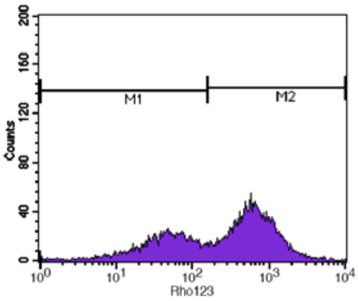

Marker	Left, Right	Events	% Gated	% Total
All	1, 9910	10000	100.00	30.28
M1	1, 155	3689	36.89	11.17
M2	155, 9910	6321	63.21	19.14

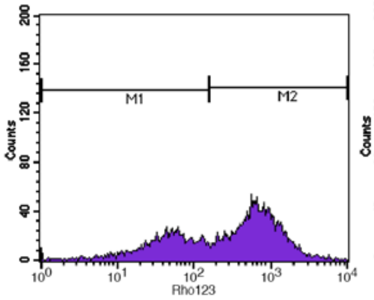

Marker	Left, Right	Events	% Gated	% Total
All	1, 9910	10000	100.00	32.13
M1	1, 155	3539	35.39	11.37
M2	155, 9910	6471	64.71	20.79

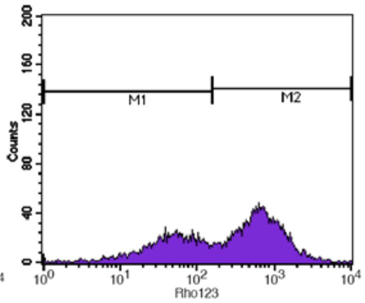

Marker	Left, Right	Events	% Gated	% Total
All	1, 9910	10000	100.00	32.08
M1	1, 155	3736	37.36	11.98
M2	155, 9910	6275	62.75	20.13
