# Supplementary material for: Grape seed proanthocyanidin extract protects lymphocytes against histone-induced apoptosis
Source: PeerJ. 2017 Mar 21;5:e3108. doi: 10.7717/peerj.3108 (PMC5363264; doi:10.7717/peerj.3108)

Bcl-2: Control, Histones, GSPE, Histones + GSPE

GAPDH

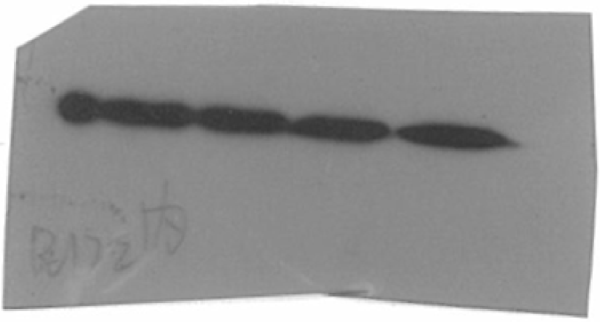

Bcl-2

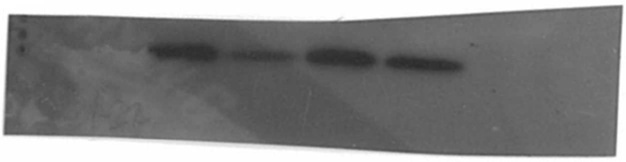

GAPDH

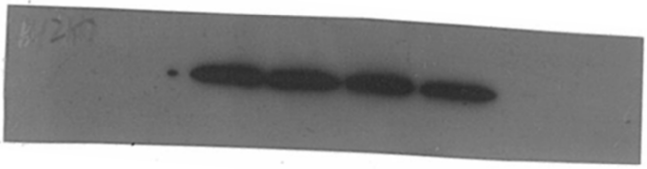

Bcl-2

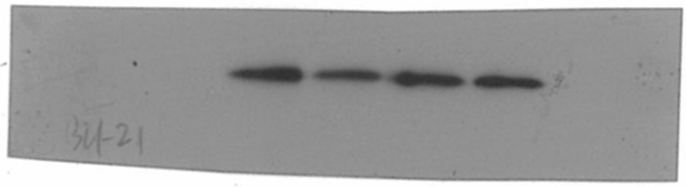

Bcl-2: Control, GSPE, Histones, Histones + GSPE

GAPDH

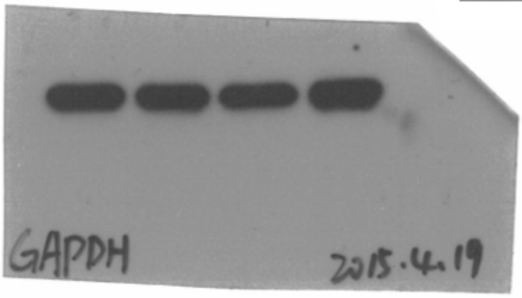

Bcl-2

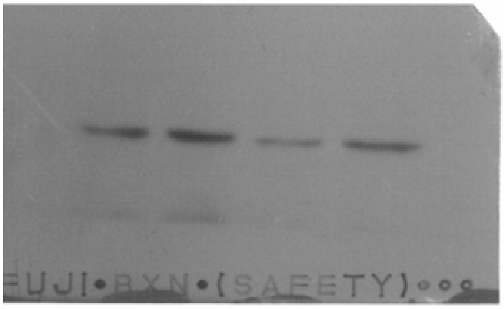

Supplement: Supplemental Information 4 [file peerj-05-3108-s004.pdf]
